# Improved Survival After Heart Failure: A Community‐Based Perspective

**DOI:** 10.1161/JAHA.113.000053

**Published:** 2013-06-21

**Authors:** Samuel W. Joffe, Kristy Webster, David D. McManus, Michael S. Kiernan, Darleen Lessard, Jorge Yarzebski, Chad Darling, Joel M. Gore, Robert J. Goldberg

**Affiliations:** 1 Department of Medicine, University of Massachusetts Medical School, Worcester, MA (S.W.J., K.W., D.D.M.M., M.S.K., J.Y., J.M.G.); 2 Department of Quantitative Health Sciences, University of Massachusetts Medical School, Worcester, MA (S.W.J., D.D.M.M., D.L., J.M.G., R.J.G.); 3 Department of Emergency Medicine, University of Massachusetts Medical School, Worcester, MA (C.D.); 4 Department of Medicine, Tufts University School of Medicine, Boston, MA (M.S.K.)

**Keywords:** acute heart failure, population surveillance, survival, time trends

## Abstract

**Background:**

Heart failure is a highly prevalent, morbid, and costly disease with a poor long‐term prognosis. Evidence‐based therapies utilized over the past 2 decades hold the promise of improved outcomes, yet few contemporary studies have examined survival trends in patients with acute heart failure. The primary objective of this population‐based study was to describe trends in short‐ and long‐term survival in patients hospitalized with acute decompensated heart failure (ADHF). A secondary objective was to examine patient characteristics associated with decreased long‐term survival.

**Methods and Results:**

We reviewed the medical records of 9748 patients hospitalized with ADHF at all 11 medical centers in central Massachusetts during 1995, 2000, 2002, and 2004. Patients hospitalized with ADHF were more likely to be elderly and to have been diagnosed with multiple comorbidities in 2004 compared with 1995. Over this period, survival was significantly improved in‐hospital, and at 1, 2, and 5 years postdischarge. Five‐year survival rates increased from 20% in 1995 to 29% in 2004. Although survival improved substantially over time, older patients and patients with chronic kidney disease, chronic obstructive pulmonary disease, anemia, low body mass index, and low blood pressures had consistently lower postdischarge survival rates than patients without these comorbidities.

**Conclusion:**

Between 1995 and 2004, patients hospitalized with ADHF have become older and increasingly comorbid. Although there has been a significant improvement in survival among these patients, their long‐term prognosis remains poor, as fewer than 1 in 3 patients hospitalized with ADHF in 2004 survived more than 5 years.

## Introduction

Heart failure (HF) is a highly prevalent, morbid, and costly disease, affecting more than 6.6 million Americans and causing more than 275 000 deaths annually.^1^ Prior to the mid‐1990s, fewer than 1 in every 5 patients hospitalized with acute decompensated heart failure (ADHF) survived more than 5 years.^1–3^ Over the past 2 decades, however, there have been numerous advances in the medical and nonpharmacologic treatment of patients with chronic HF. The use of these evidence‐based therapies has increased rapidly following the publication of the American College of Cardiology Foundation/American Heart Association (ACC/AHA) Clinical Practice Guidelines for Congestive Heart Failure in 1995.^4^ Despite encouraging improvements in the management of patients with HF, there are limited published data, especially from the more generalizable perspective of a community‐wide investigation, describing changing trends in the in‐hospital and long‐term survival of patients hospitalized with ADHF.

The primary objective of this population‐based observational study was to describe trends in short‐ and long‐term survival in patients hospitalized with ADHF in 4 study years between 1995 and 2004 in a large central New England community. A secondary study objective was to examine factors associated with decreased survival after hospital discharge for ADHF.

## Methods

Data from the Worcester Heart Failure Study were utilized for this investigation. The Worcester Heart Failure Study is a population‐based study of residents of the Worcester, MA, metropolitan area (2000 census=478 000) hospitalized with ADHF at all 11 central Massachusetts medical centers.^5–7^ These medical centers include 2 large, tertiary care academic medical centers and 9 small to midsize community hospitals. This study was approved by the Institutional Review Board at the University of Massachusetts Medical School.

The study sample consisted of greater Worcester adults hospitalized for possible ADHF during the 4 study years of 1995, 2000, 2002, and 2004. These study years were selected to coincide with population census estimates and based on the availability of federal grant support. Trained physicians and nurses performed a standardized review of the medical records of greater Worcester residents hospitalized at all 11 medical centers in central Massachusetts with primary or secondary International Classification of Disease (ICD)‐9 codes consistent with the presence of possible HF.^5–7^ A discharge diagnosis of HF (ICD‐9 code 428) was the principal diagnostic category reviewed. In addition, the medical records of patients with discharge diagnoses of hypertension, renal disease, acute cor pulmonale, cardiomyopathy, pulmonary congestion, acute lung edema, and respiratory abnormalities were reviewed to identify patients who may also have had new onset HF.^5–7^

The diagnosis of ADHF in greater Worcester residents presenting to all area hospitals with signs and symptoms of HF was confirmed based on the Framingham criteria, requiring the presence of 2 major criteria (e.g., rales, distended neck veins) or 1 major and 2 minor (e.g., cough at night, dyspnea on ordinary exertion) criteria.^8^ Patients who developed ADHF during admission for another acute illness (e.g., acute myocardial infarction) or following an interventional procedure were excluded.

Data on patient demographics, medical history, clinical characteristics, presenting symptoms, physical examination findings, test results, medications, implantable devices, and other therapies were collected. A statewide review of death certificates, the Social Security Death Index, and review of hospital medical records at participating medical centers for subsequent hospitalizations or medical care contacts was carried out to ascertain the posthospital discharge survival status of study patients.

### Data Analysis

We examined differences in the characteristics of patients in each of our 4 study years using ANOVA and Mantel‐Haenszel chi square tests for continuous and discrete variables, respectively. The Mantel‐Haenszel chi square test was used to examine the statistical significance of trends in changing patient characteristics during the years under study (Table 1).

**Table 1.** Demographic and Clinical Characteristics of Patients Hospitalized With Acute Decompensated Heart Failure

	Total Population (n=9748)	1995 Cohort (n=1949)	2000 Cohort (n=2587)	2002 Cohort (n=2743)	2004 Cohort (n=2469)	*P*‐Value for Trend*
Age (mean, y)	76.2 (±12.1)	75.7 (±11.0)	76.3 (±12.3)	76.4 (±12.3)	76.2 (±12.6)	0.19
Age (y), %						
<65	15.6	14.3	15.3	16.0	16.7	<0.01
65 to 74	21.5	26.9	20.0	19.8	20.8	
75 to 84	37.1	37.5	38.7	38.0	34.1	
≥85	25.7	21.4	27.0	26.2	28.4	
Male	43.9	42.9	43.3	42.7	46.9	<0.05
White	93.8	96.8	93.8	93.1	92.3	<0.001
Incident event	29.0	26.1	24.9	28.6	36.4	<0.001
Body mass index, kg/m^2^	27.7 (±7.6)	26.8 (±7.3)	27.6 (±7.5)	28.3 (±8.0)	28.1 (±7.4)	<0.001
Cholesterol, mg/dL	162.5 (±46.0)	175.6 (±49.0)	166.9 (±48.1)	159.8 (±42.6)	150.4 (±41.4)	<0.001
Systolic blood pressure, mm Hg	142.7 (±32.1)	145.9 (±32.5)	143.2 (±31.6)	141.8 (±32.1)	140.5 (±32.1)	<0.001
Diastolic blood pressure, mm Hg	74.7 (±19.1)	79.1 (±18.4)	75.1 (±19.0)	73.2 (±19.3)	72.5 (±19.1)	<0.001
Creatinine, mg/dL	1.64 (±1.26)	1.57 (±1.13)	1.63 (±1.21)	1.59 (±1.13)	1.77 (±1.51)	<0.001
Estimated GFR, mL/min per 1.73 m^2^	50.9 (±23.7)	51.8 (±23.2)	50.9 (±23.5)	51.5 (±23.9)	49.4 (±23.9)	<0.01
Blood urea nitrogen, mg/dL	34.4 (±25.8)	33.3 (±23.7)	35.2 (±25.4)	33.5 (±23.5)	35.5 (±30.2)	<0.005
Serum sodium, mmol/L	137.4 (±5.5)	137.7 (±5.1)	136.9 (±5.7)	137.0 (±5.7)	138.1 (±5.5)	<0.001
Glucose, mg/dL	158.5 (±69.3)	165.6 (±75.9)	161.5 (±69.8)	155.9 (±66.2)	152.5 (±66.1)	<0.001
Ejection fraction (EF), %	45.4 (±16.6)	41.7 (±14.9)	44.4 (±16.0)	46.6 (±17.0)	46.9 (±17.0)	<0.001
EF ≥50%	49.5	34.6	47.8	53.3	53.9	<0.001
EF <40%*	36.8	45.5	38.0	34.4	34.4	<0.001
LOS, days	6.1 (±7.8)	7.4 (±8.1)	5.5 (±6.6)	6.0 (±7.6)	6.1 (±8.7)	<0.001
Medical history, %						
Anemia	24.6	21.9	24.6	24.6	26.7	<0.001
Coronary heart disease	56.0	57.0	56.7	54.8	55.7	0.23
Chronic lung disease	35.9	35.5	34.3	37.9	35.7	0.37
Diabetes	39.0	39.7	39.9	37.5	39.3	0.39
Hypertension	68.7	62.3	67.3	70.7	72.8	<0.001
Peripheral vascular disease	19.7	20.2	16.7	20.9	21.0	0.22
Renal failure/Disease	25.9	21.5	24.6	25.5	31.4	<0.001
Stroke	13.2	14.3	14.8	12.9	11.5	<0.01
Symptoms, %						
Angina/Chest pain	31.2	31.2	30.1	33.4	29.9	0.96
Dyspnea/Shortness of breath	93.4	96.5	93.1	92.6	92.2	<0.001
Swelling	70.3	63.8	70.3	72.7	72.7	<0.001
Nausea/Vomiting	16	14.2	14.9	17.4	17.1	<0.01
Orthopnea	35.4	29.2	36.7	36.1	38.4	<0.001
Weakness	25.1	24.9	28.5	23.1	23.8	0.11
Weight gain	8.0	6.8	8.9	7.3	8.9	<0.05
Medication						
ACE inhibitors/ARB's	51.5	50.4	52.0	51.9	51.4	0.46
Aspirin	56.3	41.1	57.3	60.0	63.1	<0.001
Beta blockers	55.3	26.2	51.8	62.5	73.7	<0.001
Calcium channel blockers	32.7	40.2	33.9	29.8	29.7	<0.001
Lipid lowering agents	27.3	5.8	21.0	32.8	44.9	<0.001
Diuretics	97.1	97.9	97.7	96.6	96.4	<0.01
Digoxin	43.5	55.0	47.2	41.2	33.3	<0.001

Data are given as percentages or mean±SD unless otherwise noted. GFR indicates glomerular filtration rate; LOS, length of stay; ACE, angiotensin‐converting enzyme; ARB, angiotensin receptor blocker.

* Mantel‐Haenszel chi square test for trend.

* Missing in 63% of cases.

In‐hospital and 30‐day case‐fatality rates (CFRs) were calculated in a standard manner. We used a life‐table approach to examine all‐cause mortality patterns in greater Worcester residents discharged from all central Massachusetts medical centers after hospitalization for ADHF. Multivariable logistic regression analysis was used to examine changes over time in in‐hospital and 30‐day CFRs while controlling for several factors of prognostic importance. A Cox proportional hazards regression approach was utilized to identify demographic, medical history, laboratory, and clinical factors associated with a poor prognosis after hospital discharge for ADHF during the 5 year post discharge follow‐up period while controlling for potentially confounding factors and duration of follow‐up. Multivariable adjusted hazards ratios (HR) and accompanying 95% confidence intervals (CI) for factors associated with a poor prognosis after hospital discharge were calculated. In all calculated odds ratios and hazards ratios in our regression models, 1995 served as the referent year (Tables 2 and 4). Clustering within hospitals was accounted for in all regression models by using the surveylogistic procedure in SAS where hospital was specified as a cluster to avoid the underestimation of standard deviations and standard errors. Proportionality assumptions for the Cox regression model were tested with the goodness‐of‐fit test and were met for all analyses. We utilized commonly used cutpoints to categorize a number of the physiologic and laboratory values examined, as opposed to expressing these factors as continuous variables, to make the results more clinically meaningful and interpretable.

Since we did not have information available about patients' clinical or treatment status after hospital discharge, we focused our analysis on factors associated with long‐term survival that could be identified during the index hospitalization. In our multivariable adjusted regression analyses, we did not control for the use of in‐hospital therapies due to the nonrandomized observational nature of this study and potential for confounding by treatment indication. Furthermore, we did not include treatment variables in our regression models due to the difficulty in interpretation of any findings. Since ejection fraction (EF) data were assessed in only one‐third of study patients during their index hospitalization, we did not stratify our results according to EF findings or by type of HF (preserved or reduced EF).

## Results

### Baseline Characteristics

A total of 9748 metropolitan Worcester residents were discharged from all central Massachusetts medical centers with independently confirmed ADHF in 1995, 2000, 2002, and 2004. The average age of this population was 76 years, 94% were white, 56% were women, and 29% were hospitalized with HF for the first time (incident cases) (Table 1). Overall, patients with ADHF had a high burden of comorbid cardiovascular and noncardiovascular diseases including hypertension (69%), coronary artery disease (56%), diabetes (39%), chronic obstructive pulmonary disease (36%), and chronic kidney disease (26%).

Compared with patients presenting to greater Worcester hospitals in 1995, patients hospitalized with ADHF during more recent study years were older, more likely to be male, non‐white, and to present with an incident (initial episode) HF event (Table 1). These patients were also more likely to be heavier and to have lower serum cholesterol, blood pressure, estimated glomerular filtration rate findings, and serum glucose levels, but higher serum levels of blood urea nitrogen and sodium. Patients hospitalized in the most recent study years had, on average, a shorter hospital stay, and, in the more limited sample of patients with data available on EF findings, there was a trend toward increasing average EF results during the years under study. Patients hospitalized in more recent study years were also more likely to have a history of anemia, hypertension, chronic kidney disease, and stroke. Patients presenting with an initial episode of ADHF had fewer comorbidities and symptomatic complaints compared with the total study population.

In examining changing trends in hospital treatment practices during the years under study, patients hospitalized during recent study years were more likely to have been treated with aspirin, beta blockers, and lipid lowering agents; on the other hand, patients were less likely to have been treated with calcium channel blockers and digoxin during more recent as compared with earlier study years (Table 1). Relatively similar trends were observed with regards to the prescribing of these medications at the time of hospital discharge (data not shown).

### In‐Hospital and 30‐Day Case‐Fatality Rates

In‐hospital and 30‐day case fatality rates for all hospitalized patients, and separately for incident (initial) cases of ADHF, are shown in Table 2. During the period under study, a total of 704 (7.2%) patients died during their index hospitalization while 752 (8.3%) patients died within 30 days after hospital discharge. In 1995, 8.5% of patients admitted for ADHF died during hospitalization with inconsistent declines in hospital death rates noted between 2000, 2002, and 2004 (Table 2). In 1995, 8.5% of patients died within 30 days following hospital discharge for ADHF compared with 7.1% in 2004. Similar, albeit inconsistent, declining trends in in‐hospital and 30‐day all‐cause mortality rates were also observed in patients with an initial episode of ADHF.

**Table 2.** Changes Over Time in the Odds of In‐Hospital and 30‐Day CFR in Patients Hospitalized With Acute Decompensated Heart Failure

Study Year	In‐Hospital CFR% (n)	Crude Odds Ratio	Age and Sex Adjusted Odds Ratio	Multivariable Adjusted Odds Ratio*	30‐Day CFR% (n)	Crude Odds Ratio	Age and Sex Adjusted Odds Ratio	Multivariable Adjusted Odds Ratio*
Total population								
1995*(n=1949)	8.5 (166)	1.0	1.0	1.0	8.5 (152)	1.0	1.0	1.0
2000 (n=2587)	5.5 (142)	0.62 (0.53 to 0.74)*	0.61 (0.52 to 0.71)	0.66 (0.59 to 0.72)	9.4 (229)	1.11 (0.83 to 1.48)*	1.07 (0.82, 1.39)	1.05 (0.82 to 1.34)
2002 (n=2743)	8.3 (227)	0.97 (0.80 to 1.17)	0.95 (0.77 to 1.16)	1.00 (0.78 to 1.30)	8.3 (208)	0.96 (0.81 to 1.15)	0.93 (0.80, 1.07)	0.90 (0.76 to 1.05)
2004 (n=2469)	6.8 (169)	0.79 (0.69 to 0.90)	0.77 (0.66 to 0.90)	0.72 (0.61 to 0.84)	7.1 (163)	0.82 (0.59 to 1.14)	0.78 (0.61, 1.00)	0.73 (0.50 to 1.05)
Incident (newly diagnosed) cases only								
1995* (n=508)	7.9 (40)	1.0	1.0	1.0	6.6 (31)	1.0	1.0	1.0
2000 (n=645)	5.7 (37)	0.71 (0.42 to 1.21)*	0.71 (0.42 to 1.20)	0.84 (0.50 to 1.42)	7.9 (48)	1.21 (0.85 to 1.73)*	1.16 (0.78, 1.74)	1.09 (0.83 to 1.44)
2002 (n=783)	9.8 (77)	1.26 (0.86 to 1.86)	1.27 (0.87 to 1.85)	1.37 (1.12 to 1.67)	5.5 (39)	0.83 (0.47 to 1.44)	0.80 (0.42, 1.50)	0.64 (0.41 to 1.02)
2004 (n=898)	6.8 (61)	0.86 (0.64 to 1.14)	0.84 (0.65 to 1.10)	0.74 (0.50 to 1.10)	6.3 (53)	0.96 (0.74 to 1.23)	0.91 (0.64, 1.28)	0.57 (0.35 to 0.96)

CFR indicates case‐fatality rates.

* Controlled for age, sex, race, clustering by hospital, history of anemia, coronary artery disease, chronic obstructive pulmonary disease, diabetes, hypertension, peripheral vascular disease, renal disease or stroke, estimated glomerular filtration rate, serum glucose, sodium, and blood urea nitrogen levels, systolic and diastolic blood pressure, heart rate, and length of hospital stay.

* 1995 is the referent year.

* 95% CI.

After controlling for age, sex, and all major baseline comorbidities and physiologic variables (listed in Table 1), the multivariable adjusted odds of dying during hospitalization, and within 30‐days after hospital discharge, declined over time in both the total study population and in incident cases of ADHF (Table 2). Similar, however, to what had been observed in our crude unadjusted analyses, there was an inconsistent decline in in‐hospital death rates observed during the years under study after multivariable adjustment.

### Postdischarge Survival Rates

The median follow‐up time for the study was 1.6 years. Of the 9748 patients hospitalized with ADHF in this study, 8556 (87.8%) died within the first 5 years postdischarge. At all postdischarge time points examined, the survival rate of patients hospitalized with ADHF improved appreciably between 1995 and 2004, despite the presence of an increasingly older and more comorbid patient population (Figure and Table 3). Five‐year postdischarge survival rates in 1995, 2000, 2002, and 2004 were 20%, 22%, 27%, and 29%, respectively.

**Table 3.** Post Hospital Discharge Case‐Fatality Rates At Selected Time Points in Patients With Acute Decompensated Heart Failure According to Study Year

Study Year	1 Year (%)	2 Years (%)	5 Years (%)
1995	41.4	57.4	80.3
2000	38.4	54.5	77.9
2002	35.9	46.6	72.9
2004	34.7	45.3	70.5

**Figure 1.**
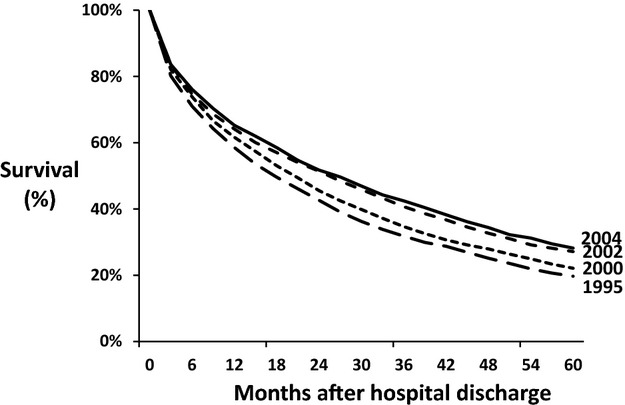
Long‐term survival after hospital discharge for acute decompensated heart failure.

Further analysis showed that survival rates improved markedly for patients who survived the first year posthospitalization for ADHF. In 2004, survival rates were 79% at 2 years postdischarge for patients that had already survived 1 year with ADHF, as compared to 52% at 2 years postdischarge for patients in the total population. A similar pattern was noted among patients with an incident episode of ADHF.

Multivariable regression models controlling for a number of different patient demographic, clustering by hospital, clinical, physiologic, and laboratory characteristics of prognostic importance (Table 1) demonstrated significantly reduced hazards rates for the risk of dying in all subsequent study years compared with 1995 (Table 4). For example, the multivariable adjusted HR for dying in 2004 was 0.63 (95% CI 0.53 to 0.74) while the HR for incident cases of HF in 2004 was 0.60 (95% CI 0.48 to 0.74) compared with patients hospitalized in the initial study year of 1995.

**Table 4.** Changes Over Time in the Crude and Multivariable Adjusted Odds of Dying After Hospital Discharge for Patients With Decompensated Acute Heart Failure

Study Year	Unadjusted Hazard Ratio	Age and Sex Adjusted Hazard Ratio	Multivariable Adjusted Hazard Ratio*
1995*	1.0	1.0	1.0
2000	0.86 (0.74 to 1.00)*	0.85 (0.75 to 0.97)	0.82 (0.75 to 0.90)
2002	0.76 (0.68 to 0.86)	0.75 (0.69 to 0.82)	0.73 (0.69 to 0.78)
2004	0.68 (0.52 to 0.88)	0.67 (0.54 to 0.83)	0.63 (0.53 to 0.74)

* Adjusted for age, sex, race, clustering by hospital, history of anemia, coronary artery disease, chronic obstructive pulmonary disease, diabetes, hypertension, peripheral vascular disease, renal disease or stroke, estimated glomerular filtration rate, serum glucose, sodium, and blood urea nitrogen levels, systolic and diastolic blood pressure, heart rate, and length of hospital stay.

* Referent year.

* 95% CI.

### Factors Associated With Decreased Survival After Hospital Discharge

A Cox proportional hazards regression analysis was conducted to identify patient characteristics independently associated with increased postdischarge mortality for both incident cases and all cases of ADHF (Table 5). This analysis showed that older age, male sex, prior HF, anemia, chronic obstructive pulmonary disease, diabetes, peripheral vascular disease, chronic kidney disease, stroke, and low blood pressures at the time of hospital presentation were associated with poorer long‐term survival. Higher serum levels of blood urea nitrogen and lower levels of serum sodium were associated with poorer long‐term survival. These factors were adversely associated with long‐term prognosis for all cases of ADHF and for incident cases with this clinical syndrome.

**Table 5.** Factors Associated With Postdischarge Mortality

Factor	All Patients		Incident Cases	
	HR	95% CI	HR	95% CI
Age, y				
<65	1.00	—	1.00	—
65 to 74	1.43	1.21 to 1.70	1.62	1.52 to 1.73
75 to 84	1.89	1.64 to 2.18	2.22	1.96 to 2.51
≥85	2.62	2.29 to 2.99	3.16	2.75 to 3.63
Male	1.06	1.02 to 1.10	1.24	1.04 to 1.47
White race	1.39	1.22 to 1.59	1.61	1.38 to 1.86
Incident case	0.74	0.65 to 0.83	—	—
Medical history				
Anemia	1.13	1.06 to 1.20	1.23	1.04 to 1.44
Coronary heart disease	1.00	0.95 to 1.06	0.99	0.93 to 1.06
Chronic obstructive pulmonary disease	1.26	1.19 to 1.34	1.48	1.35 to 1.63
Diabetes	1.08	1.01 to 1.15	1.08	0.98 to 1.18
Hypertension	0.88	0.84 to 0.92	0.89	0.77 to 1.01
Peripheral vascular disease	1.09	1.01 to 1.18	1.14	1.07 to 1.20
Renal disease	1.11	1.04 to 1.20	1.11	0.98 to 1.26
Stroke	1.19	1.14 to 1.24	1.22	1.08 to 1.38
Estimated GFR, mL/min per 1.73 m^2^				
<30	1.21	1.04 to 1.42	1.30	0.89 to 1.89
30 to 59	1.08	1.01 to 1.14	1.10	1.03 to 1.18
≥60	1.0	—	1.0	—
Serum glucose, mg/dL				
<140	1.00	—	1.00	—
140 to 199	1.00	0.95 to 1.05	0.99	0.90 to 1.09
≥200	1.02	0.91 to 1.14	1.14	0.92 to 1.41
Systolic BP, mm Hg				
<100	1.0	—	1.0	—
100 to 159	0.78	0.73 to 0.84	0.89	0.76 to 1.04
≥160	0.66	0.60 to 0.72	0.75	0.61 to 0.92
Diastolic BP, mm Hg				
<60	1.0	—	1.0	—
60 to 89	0.99	0.94 to 1.04	0.93	0.89 to 0.98
≥90	0.96	0.90 to 1.02	0.97	0.90 to 1.04
Heart rate, bpm				
<60	1.0	—	1.0	—
60 to 99	1.05	0.91 to 1.21	1.14	0.93 to 1.40
≥100	1.16	1.05 to 1.28	1.16	0.96 to 1.42
Blood urea nitrogen, mg/dL				
<43	1.0	—	1.0	—
≥43	1.45	1.30 to 1.62	1.32	0.94 to 1.86
Serum sodium, mmol/L				
<135	1.15	1.13 to 1.18	1.10	1.06 to 1.15
≥135	1.0	—	1.0	—

Referent groups: age <65 years, female sex, absence of listed comorbidities, eGFR ≥60 mL/min per 1.73 m^2^, serum glucose <140 mg/dL, systolic blood pressure <100 mm Hg, diastolic blood pressure <60 mm Hg, heart rate <60 bpm, blood urea nitrogen <43 mg/dL, and serum sodium ≥135 mmol/L. GFR indicates glomerular filtration rate; BP, blood pressure.

## Discussion

The principal finding of this community‐wide study of patients hospitalized with ADHF between 1995 and 2004 was a marked improvement in long‐term post hospital discharge survival (5‐year survival improved from 20% to 29%) during the decade‐long period under study. There were also improvements in both in‐hospital and 30‐day postdischarge survival, though these trends were somewhat inconsistent during the years under study. These improvements in survival are particularly notable given that patients admitted with ADHF have become increasingly elderly and debilitated over time. Although survival improved substantially during the years under study, the overall prognosis of patients hospitalized with ADHF remained poor, with fewer than 1 in 3 patients hospitalized in 2004 surviving more than 5 years. Older age, a history of previously diagnosed HF, low blood pressures, and a variety of comorbidities were independently associated with decreased long‐term survival.

### Study Population Characteristics

The baseline characteristics of patients hospitalized with ADHF in this study were comparable to those of patients enrolled in large HF registries in the United States and Europe, such as the Acute Decompensated Heart Failure National Registry, 2002–2004 (ADHERE), Organized Program to Initiate Lifesaving Treatment in Patients Hospitalized with Heart Failure, 2003‐2004( OPTIMIZE‐HF), and Euro Heart Failure Survey I, 2001–2002 and II, 2005 (EHFS).^9–14^ These studies have shown that patients hospitalized with ADHF are typically in their mid‐70s, a slight majority are women, and multiple cardiovascular and noncardiovascular comorbidities, including prior HF, are present.

Notably, patients admitted with ADHF in our study were significantly older and were more medically complex over time. Although the average age of our study population increased by only 0.5 years between 1995 and 2004, the proportion of patients 85 years and older increased by one‐third over time, from 21% in 1995 to 28% in 2004. Patients admitted with ADHF in 2004 were significantly more likely to have a history of chronic renal disease, hypertension, and anemia, as well as a higher body mass index, compared with those admitted in 1995. Additionally, patients admitted to central Massachusetts medical centers in 2004 were more likely to have lower serum cholesterol and glucose levels and lower blood pressures, likely due to the more extensive use of statins, antihypertensives, and antihyperglycemic medications, than patients admitted during earlier study years.^5–7^

Of interest, there was an increasing proportion of patients hospitalized with an initial episode of ADHF over time. While the reasons for this somewhat unexpected increase are unknown, this changing trend may be due to an increasing pool of patients with cardiovascular disease at risk for ADHF, changing natural history of this clinical syndrome, or to changing diagnostic or hospital admission practices during the years under study.

### Trends in In‐Hospital and 30‐Day Case‐Fatality Rates

Few studies, especially from the more generalizable perspective of a population‐based investigation, have examined trends in in‐hospital and short‐term CFR's among patients hospitalized with ADHF. In a prior cross‐sectional study, the OPTIMIZE‐HF study group reported a 3.8% in‐hospital death rate among 48 612 patients hospitalized with ADHF in 259 centers between 2003 and 2004,^10^ while a 6.7% in‐hospital CFR was observed in the EHFS, a study of 3580 patients hospitalized in 133 centers in 30 countries in the early 2000's.^12,15^ The in‐hospital death rate in EHFS was similar to the mortality rate observed in the present study. The lower in‐hospital mortality rate found in OPTIMIZE‐HF was likely related to the lower age and overall disease acuity of this patient population, as well as to a variety of other possible contributory factors.

No prior population‐based studies have assessed trends in hospital and short‐term postdischarge death rates in patients with ADHF. Our study supports previous findings from a national registry of 159 168 patients hospitalized with HF between 2002 and 2004 (ADHERE),^9^ in which multivariable‐adjusted in‐hospital CFRs declined from 4.5% to 3.1%. While somewhat higher in‐hospital CFRs were found in our population‐based study, we also observed relative declines in short‐term death rates of approximately 20% during the years under study. Our study population was on average 4 years older than patients in ADHERE, though patients in both studies had a similar profile of comorbidities.

Although differences in the duration of hospitalization or patient demographic and clinical characteristics over time could have affected trends in hospital mortality, there remained a clinically meaningful decline in the multivariable adjusted odds of dying in the hospital and at 30 days after hospital discharge between 1995 and 2004 after controlling for a number of potentially confounding factors including comorbidities, physiologic variables, and hospital length of stay. It is likely that the greater use of effective cardiac therapies, and implementation of advances in interventional procedures, have contributed to these encouraging trends. Improved patient education, discharge processes, and postdischarge care may have also contributed to reduced postdischarge mortality.

### Trends in Long‐Term Prognosis

Although the 5‐year mortality rates following hospital discharge for ADHF remained high in the present investigation, we and other research groups have shown significant improvements in long‐term survival among patients discharged from the hospital after ADHF. Our findings of improved multivariable‐adjusted postdischarge survival rates in both the total study population and in those with a first episode of ADHF are consistent with earlier observational studies in the United States^2–3^ and Scotland.^16^ The population‐based Framingham Study demonstrated a 12% decline in mortality risk per decade (1950‐1999) in study participants after the diagnosis of HF.^2^ In a cohort study from Olmsted County, MN, a decline in 5 year age‐adjusted death rates from 57% to 48% was observed among patients with a first diagnosis of HF between 1979 and 2000.^3^ In the large national Scottish cohort study of all patients admitted with a principal diagnosis of HF between 1986 and 1995, long‐term CFRs declined by 18% in men and 15% in women over the decade‐long period under study.^16^ In a prior study of the metropolitan Worcester population, we examined the long‐term prognosis of patients discharged from all greater Worcester medical centers after being hospitalized for ADHF in 1995 and 2000, finding improving trends in long‐term prognosis.^6^ The present study extends these prior findings by examining more recent study years, particularly during a time of changing HF guidelines, management practices, risk factor modification, and quality improvement initiatives.^4,17–22,13^

### Study Strengths and Limitations

The primary strengths of this study were the large, population‐based sample with independently validated hospitalization for ADHF, detailed information on patient demographic and clinical characteristics, and the comprehensive follow‐up of discharged study patients. Limitations of this study included a predominantly white population from a single region in central New England, lack of information on postdischarge events other than death, and the inability to evaluate prognosis according to type of HF (reduced versus preserved systolic function) due to limited availability of EF data for study patients. Information on cause‐specific mortality was not collected. Finally, we were unable to evaluate the potentially beneficial effects of the therapies examined in this study given the nonrandomized nature of this investigation and the potential for confounding by drug indication. We also did not collect data on the use of medications by patients on a long‐term basis after hospital discharge for ADHF.

## Conclusions

Admissions for ADHF were increasingly dominated by elderly patients with multiple comorbidities, who often required a prolonged hospital stay. Although both short‐ and long‐term survival for these patients improved significantly between 1995 and 2004, their long‐term prognosis remains poor, as fewer than 1 in 3 patients hospitalized with ADHF in 2004 survived more than 5 years. While there has been encouraging progress in the treatment and prognosis of patients hospitalized with ADHF, additional opportunity remains to improve the in‐hospital and postdischarge management of patients with this common and debilitating clinical syndrome.
